# Challenges and lessons learned from four years of planning and implementing pharmacovigilance enhancement in sub-Saharan Africa

**DOI:** 10.1186/s12889-022-13867-6

**Published:** 2022-08-17

**Authors:** Jens-Ulrich Stegmann, Viviane Jusot, Olga Menang, Gregory Gardiner, Sabino Vesce, Stephanie Volpe, Anderson Ndalama, Felix Adou, Opokua Ofori-Anyinam, Olakunle Oladehin, Yolanda Guerra Mendoza

**Affiliations:** 1 GSK, Avenue Fleming 20, 1300 Wavre, Belgium; 2 PATH, Geneva, Switzerland; 3 GSK, London, UK; 4 Present affiliations: European Medicines Agency, Amsterdam, The Netherlands; 5 GSK, Nyon, Switzerland; 6 Present affiliations: Novartis, Basel, Switzerland; 7 Pharmacy and Medicines Regulatory Authority, Lilongwe, Malawi; 8 Autorité Ivoirienne de Régulation Pharmaceutique (AIRP), Abidjan, Côte d’Ivoire; 9 GSK, Lagos, Nigeria

**Keywords:** Pharmacovigilance, Sub-Saharan Africa, Malawi, Côte d’Ivoire, Democratic Republic of Congo, Healthcare professionals, Adverse events, Pilot initiative, Training, Mentoring

## Abstract

Pharmacovigilance (PV) systems in many countries in sub-Saharan Africa (SSA) are not fully functional. The spontaneous adverse events (AE) reporting rate in SSA is lower than in any other region of the world, and healthcare professionals (HCPs) in SSA countries have limited awareness of AE surveillance and reporting procedures. The GSK PV enhancement pilot initiative, in collaboration with PATH and national PV stakeholders, aimed to strengthen passive safety surveillance through a training and mentoring program of HCPs in healthcare facilities in three SSA countries: Malawi, Côte d’Ivoire, and Democratic Republic of Congo (DRC). Project implementation was country-driven, led by the Ministry of Health via the national PV center or department, and was adapted to each country’s needs. The implementation phase for each country was scheduled to last 18 months. At project start, low AE reporting rates reflected that awareness of PV practices was very low among HCPs in all three countries, even if a national PV center already existed. Malawi did not have a functional PV system nor a national PV center prior to the start of the initiative. After 18 months of PV training and mentoring of HCPs, passive safety surveillance was enhanced significantly as shown by the increased number of AE reports: from 22 during 2000–2016 to 228 in 18 months to 511 in 30 months in Malawi, and ~ 80% of AE reports from trained healthcare facilities in Côte d’Ivoire. In DRC, project implementation ended after 7 months because of the SARS-CoV-2 pandemic. Main challenges encountered were delayed AE report transmission (1–2 months, due mainly to remoteness of healthcare facilities and complex procedures for transmitting reports to the national PV center), delayed or no causality assessment due to lack of expertise and/or funding, negative perceptions among HCPs toward AE reporting, and difficulties in engaging public health programs with the centralized AE reporting processes. This pilot project has enabled the countries to train more HCPs, increased reporting of AEs and identified KPIs that could be flexibly replicated in each country. Country ownership and empowerment is essential to sustain these improvements and build a stronger AE reporting culture.

## Background

Medicines and vaccines are approved for use after demonstrating acceptable safety and efficacy in clinical trials involving thousands of participants [1] who are carefully selected and followed up under controlled conditions for a relatively limited length of time. Because large numbers of people use these medicines after licensure over a long period of time, frequently alongside other medicines, rare or delayed adverse events (AEs) are usually detected after licensure [2]. These events, which may not be detected in the pre-licensure stage due to the limited numbers of involved individuals and follow-up time, are detected, assessed, understood and prevented through pharmacovigilance (PV), enabling evidence-based decisions on the products. An established PV system is a key pillar in effective regulatory systems. It ensures effective monitoring of the safety and efficacy of health products and that the benefit/risk profile of the products remains favorable. However, PV systems in most countries in sub-Saharan Africa (SSA) are not fully functional, with weak legal frameworks, structures, and procedures to collect and evaluate AEs related to medicinal products [3–6]. Also, healthcare professionals (HCPs) in these countries, have limited awareness of AE surveillance and reporting procedures [7–11]. Consequently, rates of AE reporting are very low in this region. Prior to October 2015, less than 1% of the cumulative number of individual case safety reports (ICSRs) in the World Health Organization (WHO) global safety database, VigiBase, came from Africa [12]. An assessment in four east African countries (Ethiopia, Kenya, Rwanda, and Tanzania) in 2018 found that ICSRs were submitted to national PV systems by a maximum of 1% of healthcare facilities (HCFs) per country [6]. Owing to these shortcomings, the training of HCPs is instrumental in reporting AEs and detecting potentially rare and unexpected AEs. Consequently, the GSK PV enhancement pilot initiative was launched in 2016–2017 in three SSA countries (Malawi [11], Côte d’Ivoire, and the Democratic Republic of Congo [DRC]) to encourage and strengthen passive safety surveillance through a training and mentoring program for HCPs, who are the frontline contact with patients, at their HCFs [11]. Raising awareness of PV among HCPs is an important first step toward building a stronger AE reporting culture [13]. Even for established products, generating regional post-marketing safety data is important because the safety profile of these products is mainly derived from high-income countries with well-established PV systems. However, that may differ from region to region due to environmental (e.g. underlying conditions such as HIV infection or malnutrition) and genetic polymorphism as well as age, sex or physiological changes [12, 14–18]. The accelerated introduction of new therapeutics and vaccines in SSA countries, such as those against the severe acute respiratory syndrome coronavirus 2 (SARS-CoV-2) or malaria, give added impetus for creating functional PV systems [14, 17, 19]. Having PV specific data for Africa enables national regulatory authorities (NRAs) to take informed decisions regarding the medicines used in the continent.

At the start of the PV enhancement initiative, Malawi did not have a functional PV system or national PV center [11]. By contrast, Côte d’Ivoire and DRC had functional national PV centers [4], with PV legislation in place, and both countries were members of the WHO Program for International Drug Monitoring (PIDM) since 2010 [20]. Nonetheless, AE reporting was very low, and awareness of PV among HCPs was poor in all three countries.

Implementation of the PV enhancement initiative was led in each country by the Ministry of Health (MoH) via the national PV center. The initiative was adapted to each country’s needs; decisions on appropriate methods for enhancing PV systems were made by national PV stakeholders from the country’s regulatory authority, MoH, and academia, in partnership with PATH and GSK. The project aimed to enhance reporting of AEs, that is suspected adverse drug reactions (ADRs) and AEs following immunization (AEFIs), during a planned implementation period of 18 months.

**Fig. 1:**
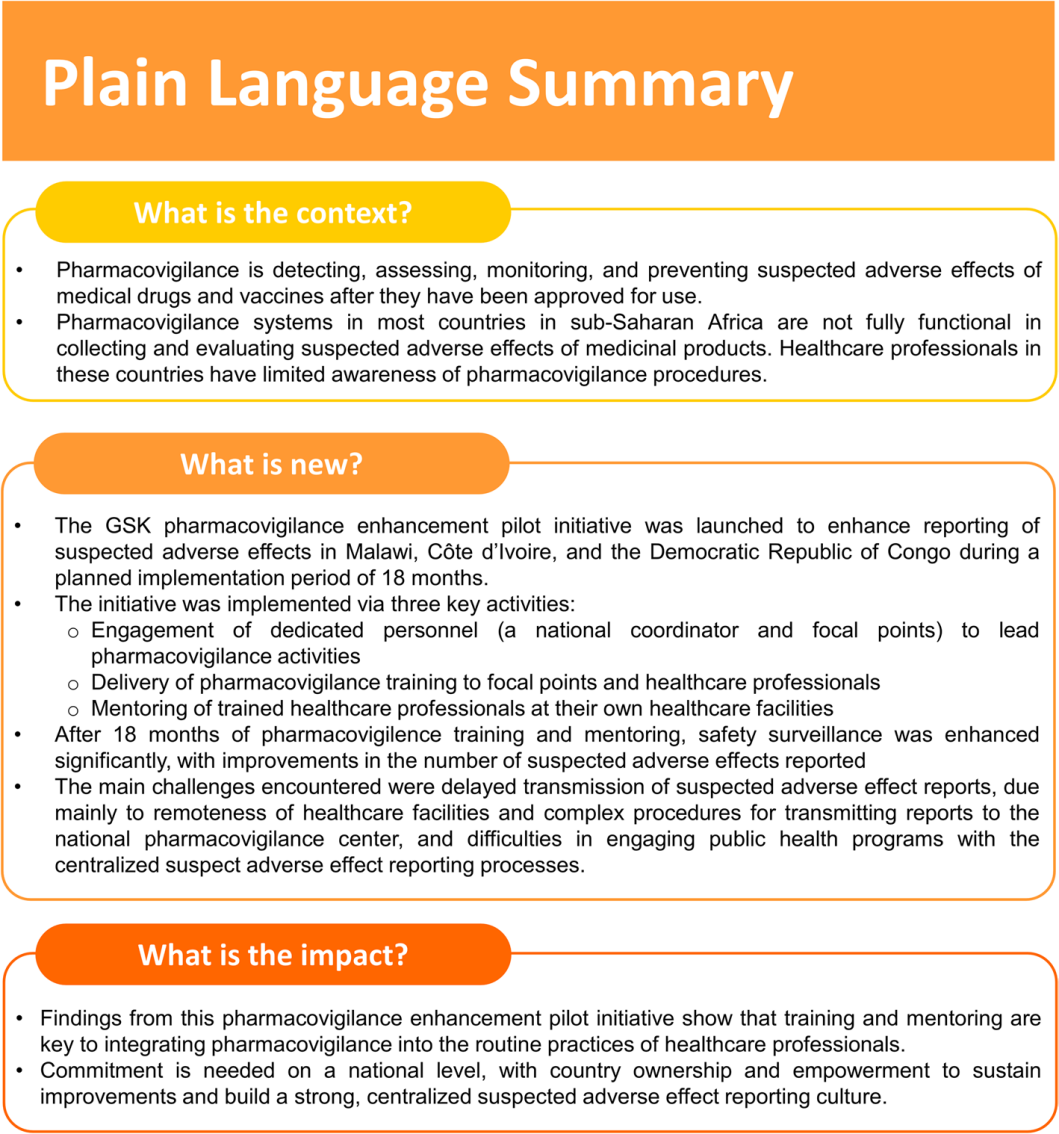
Plain language summary

In this article, we present the main challenges encountered and lessons learned during the planning and implementation phases of the PV enhancement initiative in the three SSA countries. The key aspects of this project are presented in a Plain Language Summary (Fig. 1).

### The PV enhancement pilot initiative

**Fig. 2:**
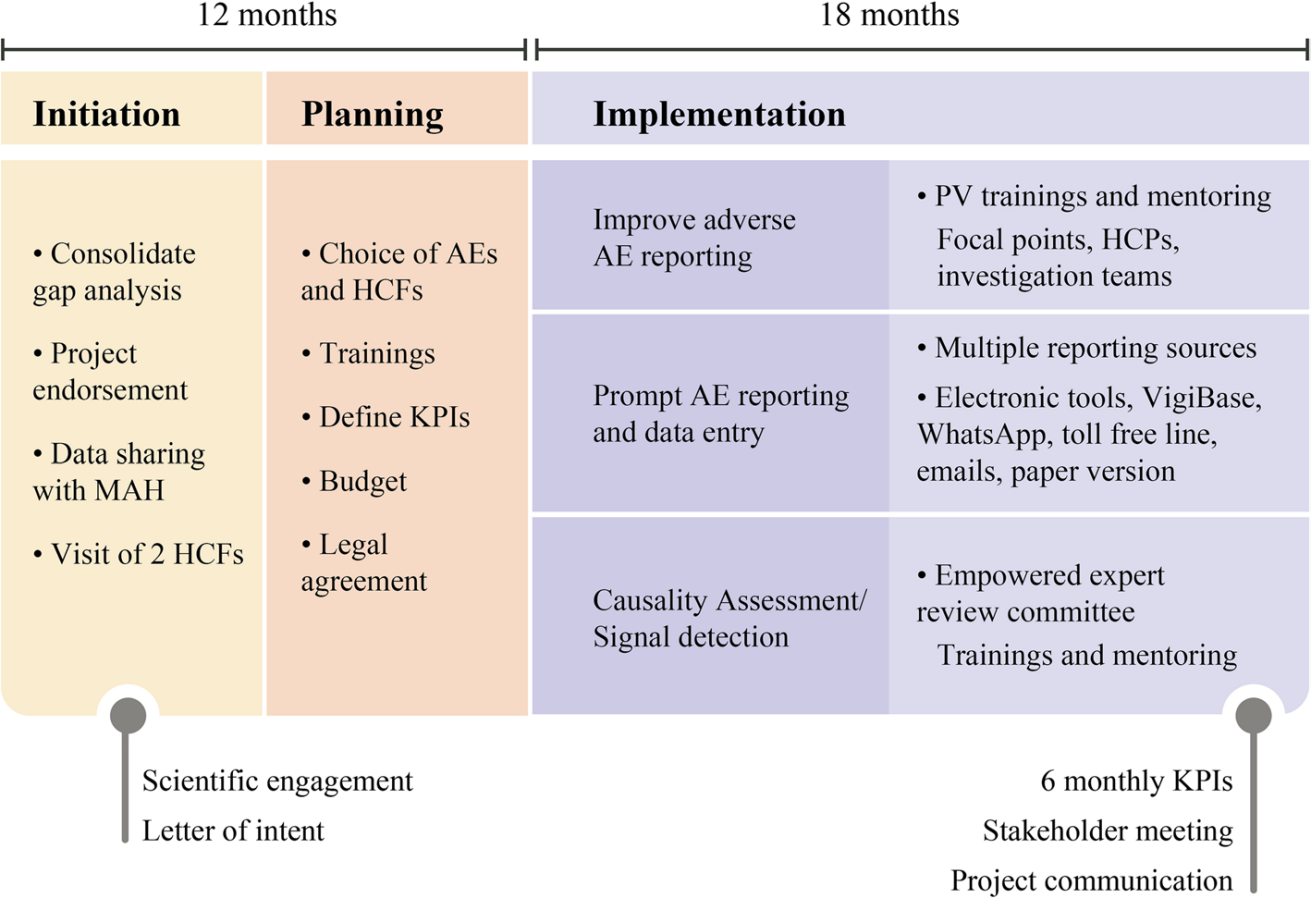
PV enhancement project road map. AE, adverse event; HCF, healthcare facility; HCP, healthcare professional; KPI, key performance indicator; PV, pharmacovigilance

The selection of SSA countries for inclusion in the PV enhancement project was based on situational and gap analyses of the national PV systems, conducted to determine the level of safety surveillance, the capabilities of the existing systems, and areas for improvement [11]. A systematic road map for implementing the project consisted of a five-step process: evaluation of the country’s PV system and gap analysis; project endorsement by national stakeholders (project initiation); project preparation; project implementation; and project evaluation. This road map (Fig. 2) has been repeated in each of the pilot countries, tailoring AE reporting to each health care facility reality.

#### Evaluation of the country’s PV system and gap analysis

The level of safety surveillance, the capabilities of the existing systems, and areas for improvement were determined by gap and situational analyses using information from literature and official documents. The project was presented to the MoH of each country, and authorization was requested to meet with national PV stakeholders (see below) and to visit HCFs for a better understanding of AE reporting and areas of improvement.

#### Project endorsement by national stakeholders (initiation)

The project was endorsed during initiation meetings (May 2016 in Malawi, November 2016 in Côte d’Ivoire, and July 2017 in DRC) with national PV stakeholders from the national PV center, academia, the NRA, Expanded Program on Immunization (EPI), MoH, and WHO. The meeting provided an opportunity to create partnerships and to openly discuss the challenges of PV in SSA and in each pilot country, thereby completing the gap analysis. The PV challenges in low- and middle-income countries (LMICs) were identified by literature search before approaching the countries. At the initiation meeting the general challenges were discussed and confirmed by the national PV stakeholders of each country. The national PV experts provided information on what the PV system had achieved, gaps within the PV system, a mapping of PV training in recent years, potential trainers, AE reporting flow chart, major hurdles and challenges for the conduct of PV, areas that needed further development and support, and other PV initiatives and funding partners in the country. Additionally, a field visit to two to three HCFs was made to observe how AE reporting is done. This enabled the project to be flexibly tailored to each country’s need and functioning.

#### Project preparation

A planning meeting was held with national PV stakeholders in each country within 6 weeks of the initiation meeting in Malawi and DRC and within 3 months in Côte d’Ivoire, during which the methodologies for enhancing each national PV system were discussed and approved. For Malawi (population: > 18 million), a nationwide training strategy was adopted because the PV system was in its very early stage. In Côte d’Ivoire, national PV stakeholders agreed to implement the project in the Abidjan region (population: 5 million), which was one of the regions lacking PV focal point training. In DRC, the approved strategy was to create three provincial PV centers at Kinshasa (population: 12 million) to improve PV coordination and transmission of AE reports to the national PV center.

The national PV stakeholders in each country defined the training and mentoring program, the target audience, and the methods of AE report transmission to the national PV center. In addition, the most relevant challenges in PV were translated into key performance indicators (KPIs) that were to be collected by each country’s national PV coordinator throughout the implementation phase and evaluated every 6 months by the stakeholders to monitor progress. Any new challenges observed were to be discussed and documented throughout the entire project period.

#### KPIs

KPIs consisted of the number and quality (availability of the 4 essential minimal information required) of AEs reported, the proportion of AE forms transmitted from the HCFs to the national PV center within 48 hours, the proportion of complete AE reports, the proportion of serious AE reports investigated, and the proportion of received AE forms entered into the PV database within two working days. An additional KPI was defined in DRC and Côte d’Ivoire because the national PV centers were better established: causality assessment of serious AEs performed by the national expert review committee. The baseline value required among the KPIs was “the number of AEs” notified prior to project implementation. The target values for the other KPIs were decided by the national PV experts based on their judgement of the functioning and maturity of their PV system. The KPIs were evaluated by the national stakeholders together with GSK every 6 months and at the end of the project, as described previously [11].

#### Project implementation

Implementation was via three key activities:

1. Engagement of dedicated personnel (national PV coordinator and PV focal points) to lead PV activities;

An 18-month implementation phase was planned. In Malawi, this was extended to 30 months to support the expansion of HCP trainings, further improve the quality of AE reporting, and data entry into VigiBase (see ‘Challenges encountered during the pilot project’). In all three countries, the PV coordinator was responsible for maintaining collaborations with national stakeholders and other partners, training and mentoring PV focal points, and for establishing PV trainings in disease control programs, such as for malaria, tuberculosis, and HIV. In Côte d’Ivoire and DRC, personnel at the national PV centers took on the coordination role. While there was one PV coordinator in Côte d’Ivoire, DRC had three coordinators responsible for each of the provincial PV centers. In Côte d’Ivoire and DRC, PV focal points were pharmacists and clinicians. In Malawi, EPI coordinators were also engaged as PV focal points because of their involvement in vaccination programs and AEFIs surveillance. Except for the national PV coordinator’s role in Malawi, no new positions were created by each country’s MoH; existing infrastructure and personnel were engaged where possible for sustainability.

2. PV trainings

Basic PV trainings to PV focal points. PV trainings consisted of an introduction (*The rationale for PV in SSA*) and 5 training modules: *PV and safety surveillance overview, Importance and impact of vaccination, AEFIs including case studies, AEFI surveillance,* and *ADRs including case studies*.

Abridged PV trainings to HCPs: PV trainings provided to HCPs at their facilities consisted of the following: Importance of detecting and reporting AEs, Identifying AE in patients, Identifying suspected AEs, Reporting suspected adverse AEs to the national pharmacovigilance Centre: which AEs to report, when to report, minimum and key information required, and Practice reporting of AEs.

In Malawi, GSK, PATH, and colleagues from the Pharmacy Department of the College of Medicine, Blantyre, provided the initial training sessions. Côte d’Ivoire and DRC already had suitable training materials and national PV stakeholders who took the lead on training the PV focal points. The abridged training to HCPs was developed by GSK and PATH, and each country was able to adapt it as appropriate. In Malawi, training sessions were held every six to 8 weeks, each time at three different HCFs (average 25 HCPs per HCF). In Côte d’Ivoire, three training sessions (involving around 75 HCPs) were provided at each selected HCF.

3. Evaluation of PV trainings

The impact of effective training could be assessed through the number of AEs reported from the trained facilities and by the quality of AE reports (having the four essential information: the medicinal product, reporter, the event and the patient).

Continuous mentoring of trained HCPs at their HCFs, through regular site visits and telephone contact from focal points, was established to integrate safety surveillance into routine clinical practices. This regular contact was important for providing HCPs with feedback on AE reports, support for management of serious AEs, and information exchange. Of note, continuous mentoring has previously demonstrated its benefits in developing a PV system in SSA [21].

### Project evaluation

Training and mentoring of HCPs enhanced PV:

In all three pilot countries, low AE reporting rates reflected that HCPs’ awareness of the importance of safety surveillance of medicines and spontaneous AE reporting before project implementation, irrespective of whether a national PV center existed (as in Côte d’Ivoire and DRC [4]) or not (Malawi). This contributed substantially to the low rate of AE reporting. During the training, awareness and consequently AE report rates increased. For instance, in Malawi, no documented trainings had been provided to HCPs and the maximum ever reported per year was 10 AEs (and 22 in total) between 2000 and 2016, and there were years with no reporting. Consequently, the country adopted as KPI to report 10 AEs per year, but in the 18 months of project implementation (from November 2016 to May 2018), 228 AEs were reported [11]. The improved rate of ICSR notification enabled Malawi to become the 135th full member of the WHO PIDM in 2019 [22]. After 30 months of implementation, more than 1000 HCPs had been trained and 511 AE reports were received at the national PV center.

In Côte d’Ivoire, 1247 HCPs were trained over 18 months (August 2018 to January 2020) and approximately 80% of the spontaneous AEs that were notified during this period were from trained facilities. In DRC, the project could not be extended as planned because of the SARS-CoV-2 pandemic, but available information received from the country prior to project termination showed that in 7 months (August 2019 to February 2020), 102 HCPs were trained and 42 AEs were notified. Additional details for Côte d’Ivoire will be published separately as data accrues.

The mentoring visits motivated HCPs to report AEs and offered the opportunity to address any challenges encountered. Engaging and communicating with hospital management and senior medical staff helped maintain their support for the training program, and they encouraged HCPs to notify safety issues through repeated reminders at routine clinical meetings.

### Challenges encountered during the pilot project

**Fig. 3:**
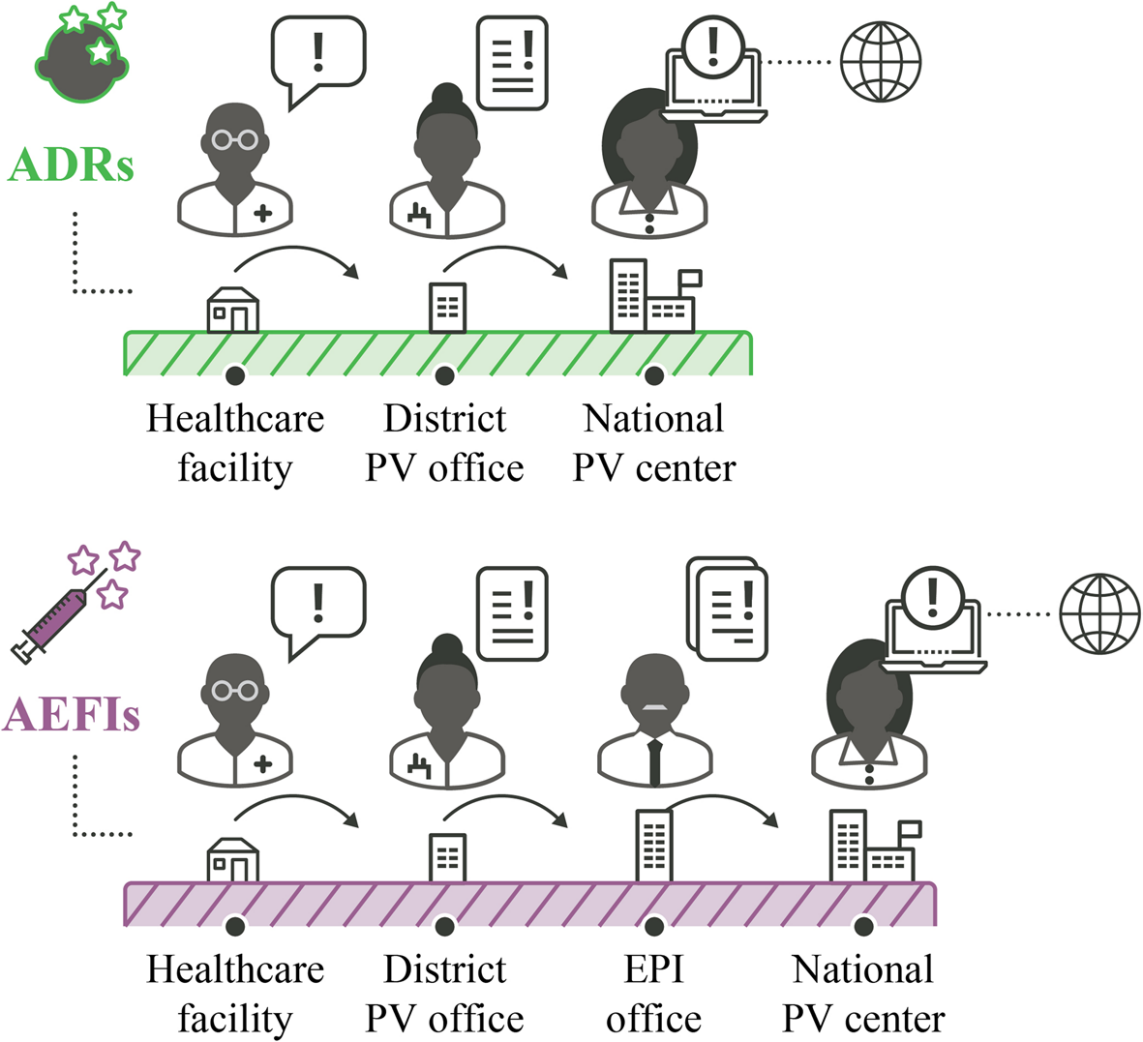
The general process for adverse event reporting in the three countries. ADR, adverse drug reaction; AEFI, adverse event following immunization; EPI, Expanded Program on Immunization; PV, pharmacovigilance

**Fig. 4:**
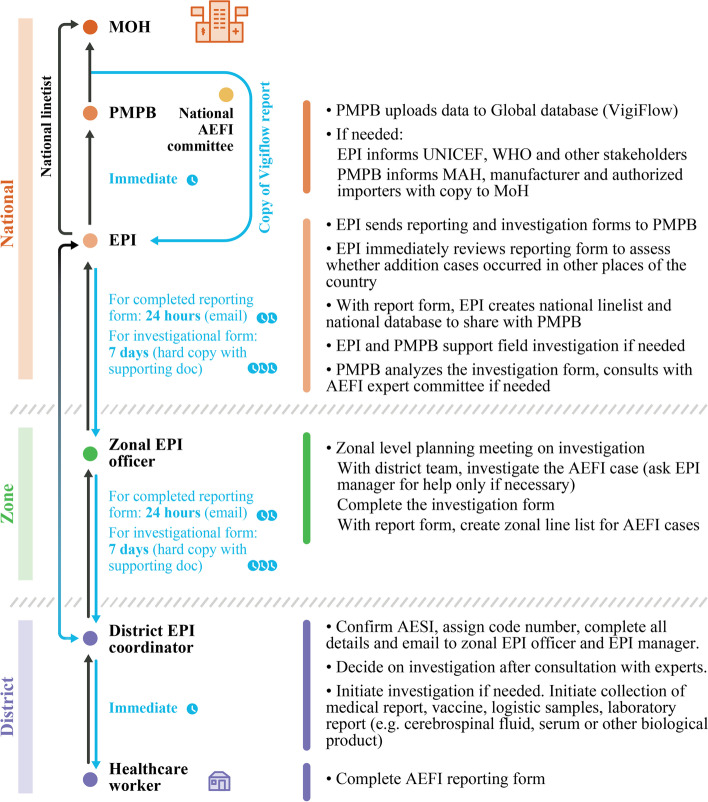
AEFI notification scheme in Malawi. AEFI, adverse event following immunization; EPI, Expanded Program on Immunization; MAH, marketing authorization holder; MoH, Ministry of health; PMPB, Pharmacy, Medicines and Poison Board (currently PMRA: Pharmacy and Medicines Regulatory Authority)

**Fig. 5:**
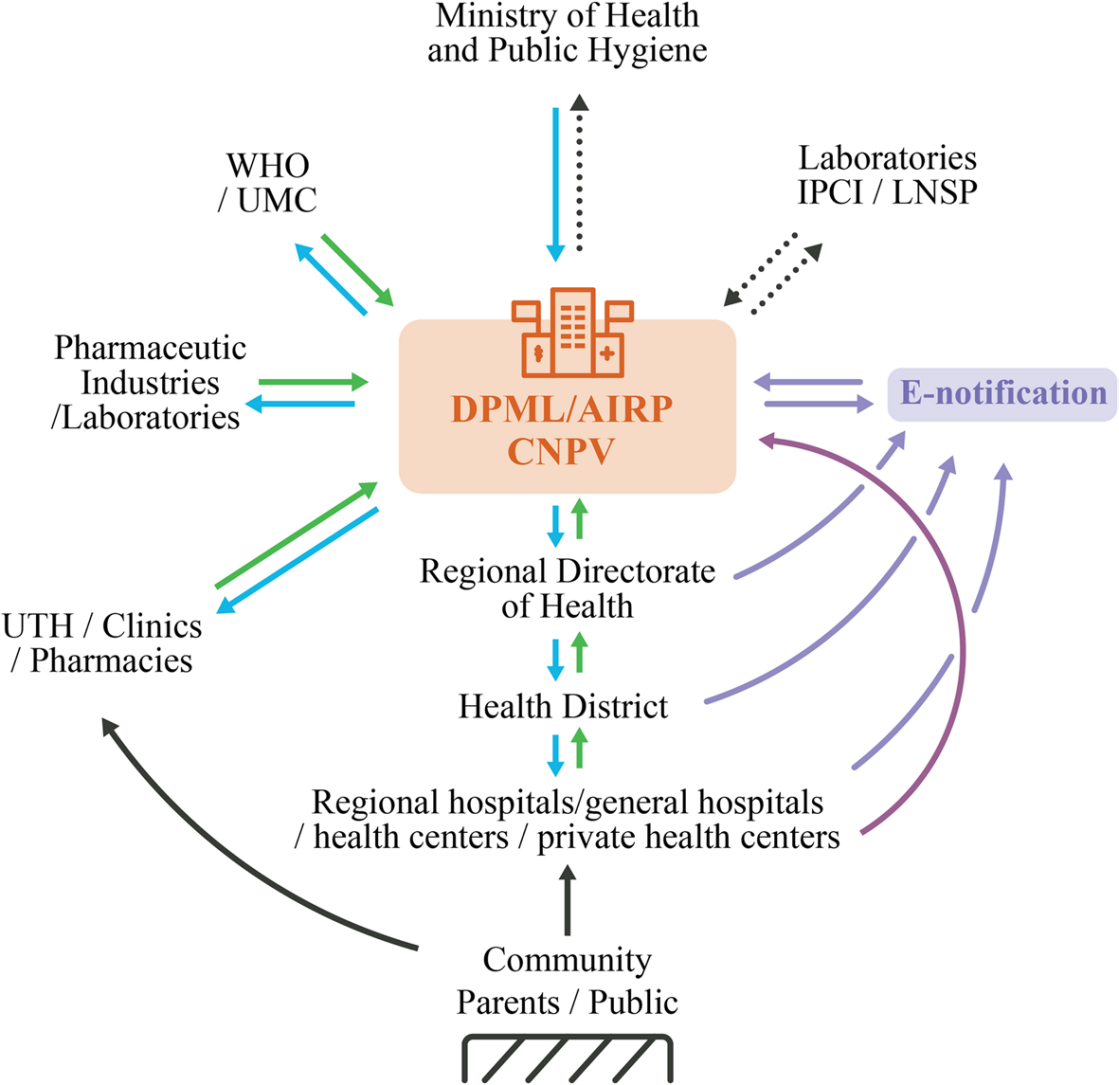
AE notification scheme in Côte D’Ivoire. AIRP, l’Agence Ivoirienne de Régulation Pharmaceutique (Ivorian Pharmaceutical Regulatory Agency); CNPV, Centre National de la Pharmacovigilance (National Pharmacovigilance Center); DPML: Direction de la Pharmacie des Médicaments et des Laboratoires (Department of Pharmacy, Medicines and Laboratories); IPCI: Institut Pasteur de Côte d’Ivoire (Pasteur Institute of Côte d’Ivoire); LNSP: Laboratoire Nationale de Santé Publique (National Laboratory for Public Health); UMC, Uppsala Monitoring Centre; UTH, University Teaching Hospital; WHO, World Health Organization

1. *Delayed transmission of AE reports from all levels of healthcare system to the national PV center*: This was observed across the three pilot countries. In Malawi, during the initial 18-month period, only two of 228 AE reports were transmitted to the national PV center within 48 hours; on average, it took one to 2 months for AE reports to be transmitted. The trend was similar in DRC and in Côte d’Ivoire during the early months of project implementation. This was partly due to the remoteness of some facilities and complex reporting pathways, which differed for suspected ADRs and AEFIs (Figs. 3, 4 and 5). AEFI reports were channeled through the EPI office to the national PV center, and reports sent via pre-paid envelopes (during the first 3 months of implementation in Malawi) were never delivered to the EPI office or national PV center, while some of the other AEFI reports were sent to the national PV center with a one- to two-month’s delay. Moreover, focal points were assigned to a particular category of AE (either suspected ADRs or AEFIs or AEs associated with traditional medicine), which made the reporting procedure even more complex for HCPs. In Côte d’Ivoire, after the first 6 months of project implementation, the focal points began to transmit the forms electronically by scanning and sending them via email from the facilities to the national PV center.
2. *Transmission and format of AE reporting forms*: AE reporting forms are available in all three countries. In Côte d’Ivoire and DRC, the same AE reporting form is used for AEFIs, ADRs and traditional medicine. Paper forms sent via the post office were unlikely to be delivered, as observed in Malawi, and regular internet connectivity issues impeded reporting via email. This was partially resolved by enabling electronic reporting of AEs via WhatsApp mobile messages. In Malawi, feedback from HCPs suggested that the three-pages long ADR reporting form was not user friendly and therefore also impeded reporting. This form was eventually replaced with a one-page form [11]. Malawi also adopted the WHO’s one-page AEFI reporting form.
3. *Negative perceptions among HCPs toward AE reporting*: As reported in other studies on PV in LMICs [7, 10], HCPs feared litigation and believed that reporting AEs such as injection site abscess could reflect negatively on their professional abilities. Others believed that AEs were normal occurrences and were not aware that they had to be reported.
4. *Delayed entry of safety data into WHO’s VigiBase at the national PV center*: This was observed across the three pilot countries, primarily due to lack of trained staff. In Malawi, a data manager was only recruited in June 2018, 20 months after project implementation began. In Côte d’Ivoire and DRC, there was no dedicated person for data entry within the national PV center, delaying entry of safety data into VigiBase.
5. *Coordination with public health programs and the EPI*: Engaging and coordinating with public health programs, such as national EPI and disease control programs, was a major challenge. In many countries in SSA, including the pilot countries, these programs have dedicated AE reporting processes, and may carry out PV activities without involving national PV centers [21]. Better collaboration and pooling of resources could improve the PV system and avoid duplicated efforts.
6. *PV personnel*: In Malawi, a national PV coordinator was only recruited 6 months into implementation and was replaced after 1 year. This negatively impacted coordination of national PV enhancement efforts and, consequently, reporting of AEs [11]. Furthermore, in Malawi, EPI coordinators, whose routine function is to coordinate vaccination campaigns, were assigned with the additional task of PV focal points and were therefore less engaged with PV activities during vaccination campaigns or epidemic periods and subject to regular transfer to other functions. Pairing an EPI coordinator with a pharmacist or physician proved to be particularly effective, especially for the success of HCP mentoring. Moreover, initial trainings targeted health surveillance agents, with insufficient medical knowledge to detect and report an AE. In DRC, project coordination and information sharing were not optimal because there was no dedicated national PV coordinator with overall responsibility for planning, implementation, and communicating progress on a timely basis.
7. *Administrative complexities*: In Côte d’Ivoire and DRC, administrative complexities in finalizing and signing the legal agreement with the country’s MoH was a major challenge during the planning phase. This delayed project implementation; the planning meeting in Côte d’Ivoire was held in February 2017 and PV trainings only began 18 months later in August 2018. In DRC, the planning meeting was held in August 2017 and implementation began almost 2 years later, in July 2019.
8. *Delay in causality assessment*: The essence of improving AE reporting is to enable any emerging signals to be detected and assessed for any causal relationship with the medicinal product. In Côte d’Ivoire, an expert review committee for causality assessment exists but never met during the 18 months due to lack of funding and coordination. In Malawi, an expert review committee did not exist at the beginning of the project but was constituted and trained on causality assessment by WHO 1 year into implementation. However, where there were safety issues that needed assessment, the committee could not meet either due to lack of funding and coordination.

The major challenges encountered as well as solutions to these are summarized in Table 1.

**Table 1:** The major challenges encountered during the PV enhancement pilot project and solutions to these

Country	Challenge	Solution
**All three countries**	Delayed transmission of AE reports from all levels of healthcare system to national PV center	• PV coordinator and focal points to be pro-active in ensuring reports are collected and reach national PV center • PV coordinator to update focal points regularly on reports received by national PV center • Partner with established organizations to assist with transmission from remote HCFs • Continuous mentoring to maintain motivation • Recognize achievements e.g. certificates to focal points with > 5 AEs reported from district • Enable electronic reporting of AEs via mobile phone messenger applications
	ADR reporting form not standard among countries and not as user-friendly as 1-page WHO AEFI reporting form	• Following consultation with national experts, 1-page ADR form introduced in Malawi, in line with 1-page and 1.5-page forms in Côte d’Ivoire and DRC
	Perceptions among HCPs toward AE reporting	• Emphasize importance of reporting procedure • Reassure HCPs that AEs can occur even when medicine has been used correctly • Engage hospital management and involve senior staff in PV training
	Delayed data entry into VigiBase	• Engage data manager within national PV center with clear roles and responsibilities
	PV-related activities ongoing within public health programs without knowledge of key PV stakeholders and PV coordinator; full collaboration with EPI not established	• Present PV enhancement plan to program directors • Gain participation of PV coordinator in PV trainings organized by health programs
	ERC not sufficiently trained (Malawi) and no regular causality assessment meetings following signal detection (all three countries)	• Allocate funding to ensure routine functioning of ERC on causality assessment • PV coordinator to ensure safety concerns are submitted to ERC and that meetings are organized promptly
**Malawi**	No official PV coordinator until six months into implementation; replaced after one year	• Plan for and engage PV coordinator ahead of implementation to allow for continuity • Train the national PV center personnel in AE data entry into VigiFlow for sharing into VigiBase
	EPI coordinators engaged as focal points: tended to become less concerned with PV during vaccination campaigns or epidemics; regularly transferred to other functions	• Engage clinicians and pharmacists as focal points • Engage back-ups to PV coordinator and focal points
	Initial training focused on HSAs who had limited medical knowledge to complete AE reports	• Train wide range of HCPs: HSAs, clinicians, nurses, pharmacists, etc.
**Côte d’Ivoire and DRC**	Delayed project implementation because of administrative complexities	• Anticipate delays in project timelines • Examine all possible constraints with approving legal agreement • Address issues that may delay implementation • Re-present project to newly appointed PV stakeholders in country

*ADR* Adverse drug reaction, *AE* Adverse event, *AEFI* Adverse event following immunization, *DRC* Democratic Republic of Congo, *EPI* Expanded Program on Immunization, *ERC* Expert review committee, *HCF* Healthcare facility, *HCP* Healthcare professional, *HSA* Health surveillance agent, *PV* Pharmacovigilance, *WHO* World Health Organization

### Lessons learned and recommendations for PV enhancement in LMICs

#### Realistic planning and implementation timelines

An implementation phase of 18 months was insufficient to strengthen AE reporting and instill PV as a routine HCP practice in LMICs with immature PV systems. In this context, we recommend a project implementation period of at least 3 years, with a project preparation period of 6–12 months. During the planning phase, it is also crucial to map stakeholders and ongoing PV interventions, and define feasible and sustainable areas for improvement.

In Côte d’Ivoire, implementation was well structured, largely because of the efforts of the PV coordinator from the beginning, the well-planned training and mentoring schedule, and effective collaborations between academia, hospital administration, and the PV center in PV trainings and project follow-up. This highlights the importance of synergy among national stakeholders for effective PV enhancement. It may also be beneficial to adopt a stepwise approach to PV training within each country, either by region or by levels of healthcare system, depending on the gaps that exist in the PV system. Flexible adaptation of the project may facilitate better results over the long term and easier roll-out in other regions or HCFs.

#### Cost-effective and user-friendly methods

The project was successful in terms of the number of HCPs trained and the subsequent increase in AE reporting [11]. In-house training sessions provided by national PV stakeholders that used qualified trainers from the national PV department or NRA, EPI, and academia were a cost-efficient alternative to outsourcing. Engagement of HCPs already involved with AE reporting to reinforce PV training is another cost-effective recommendation.

Adopting electronic reporting tools that are compatible with the national database and VigiBase should be prioritized to capture the information needed for signal detection and to facilitate data sharing. The HCPs should be trained accordingly on how to use the tool(s). Unavailable, unreliable, or expensive internet access is a key barrier to electronic transmission, as identified in our project and in evaluations of electronic PV reporting systems in other SSA settings [11, 23]. This is likely to improve over time, with better internet coverage in LMICs and increased use of smartphones and tablets for safety surveillance [24]. Where electronic transmission is not feasible, the adoption of user-friendly suspected ADR and AEFI reporting forms is likely to encourage PV as a routine practice by HCPs [13]. Internal partnerships with institutions that reach out to remote facilities on a weekly basis should be established to enable paper AE reports to be transmitted promptly to the national PV center for timely signal detection. Côte d’Ivoire is currently using e-notification through the Med Safety App for direct and prompt transfer of the AEFI reports into VigiBase.

Expert review committees should be empowered with expertise on qualitative and quantitative assessments as well as funding to meet for prompt signal detection whenever there is an identified issue.

#### Choice of dedicated implementation personnel

Our experience suggests an advantage in having clinicians and pharmacists as PV focal points because they have the medical knowledge to detect and notify AEs. Additionally, clinicians are likely to have a motivating influence within the healthcare system hierarchy. It is also important that focal points are responsible for reporting all AEs: AEFIs, suspected ADRs, and traditional medicine-related AEs. The ideal candidate for a PV coordinator should have a medical background and be trained on PV, and there should be a back-up plan in place for training and mentoring.

In Côte d’Ivoire, the PV coordinator had adequate knowledge and field experience in PV, thereby facilitating coordination of national PV activities. Also, national PV stakeholders from academia, EPI, and the PV center pooled their expertise and delivered training sessions to focal points, using a mixture of lectures, case studies, and practice AE reporting sessions, and encouraging information sharing among the focal points. In DRC, the expert in-house PV trainings were effective, as provided by PV stakeholders from the national PV center, EPI, and Division of Pharmacy and Medicine.

#### Buy in and leadership from MoH

For many PV projects associated with public health programs in SSA, as soon as external funding from development partners ceases, PV stops or stalls because PV enhancement is not seen as a priority, and, due to the limited MoH budget, no funding is allocated to this activity [4]. Maintaining a functional PV program requires national funding and strategies to encourage HCPs to report AEs. These may range from formal letters to HCFs to the development of clinical guidelines that position AE reporting as a routine task for HCPs. PV should be included in the agendas of routine HCF meetings, ensuring that HCPs have time allocated to participate, and should be integrated in the curricula of medical, nursing, and pharmacy schools. Engagement of professional medical associations in PV enhancement activities would provide another avenue for PV training and for reaching out to many HCPs. Discussions held with PV focal points in the different pilot countries indicated the necessity of providing sitting fees to HCPs each time trainings are held. Considering the limited budget allocated to PV, this could hamper sustainability of follow-up meetings and PV activities by the country’s MoH.

Additionally, at the national level, the countries are advised to have established PV guidelines and regulations, that could further reinforce the importance of HCP contribution to AE reporting as well as highlight procedures on safety reporting and signal detection. Collaborations with institutions such as WHO, non-governmental organizations like PATH, that have extensive expertise on drafting PV guidelines should be emphasized. The importance of country commitment to providing PV trainings and increasing AE reporting has been shown in Eritrea, where more than 95% of all HCPs were sensitized, consequently achieving maturity level three on the WHO rapid benchmarking assessment [25].

#### Central coordination of national PV activities

The national PV center needs to be the central PV coordinating body for all PV activities and AE reporting nationwide. Effective and transparent collaboration with the EPI is crucial in addition to conducting PV in collaboration with public health programs or development partners. We therefore recommend that the person entrusted with national PV coordination has an integral role within the national PV center. The PV coordinator should identify EPI and public health programs and engage them in the centralized safety surveillance of medicines by presenting the PV enhancement plan to program directors. A lack of engagement with centralized AE reporting processes by public health programs hampers an integrated approach toward PV and comprehensive reporting of AEs nationally [4].

The project has employed a systematic road map for PV enhancement in the selected countries, which could be sustainable and customized to each country and HCF reality and needs. During the 30 months of project implementation, Malawi notified 511 AEs into VigiFlow. Post implementation, the country currently has more than 1000 ICSRs notified into VigiFlow to date. Côte d’Ivoire has notified more than 3000 ICSRs into VigiBase from February 2020 to date. This indicates that, following the AE reporting awareness raised through the PV enhancement project, efforts and other projects from different stakeholders have helped with the continuity of the PV system improvement in those countries.

## Conclusions

Considering the limited awareness and poor resources for PV in SSA countries, findings from this PV enhancement pilot initiative have shown that safety monitoring of medicinal products should start at the HCP level. This can be achieved through continuous trainings and mentoring, which are key to raising awareness of PV among HCPs, improving spontaneous AE reporting, and integrating PV into the routine practices of HCPs.

The PV enhancement pilot project has been successful to date as it has enabled the countries to train more HCPs, increased reporting of AEs and identified KPIs that could be flexibly replicated in each country based on the maturity of their PV systems. Publishing our project experience is aimed to raise awareness, with a systematic road map model for implementation and KPIs that are crucial for other LMICs to see what had been done. Sharing the challenges allows countries to adapt the project and set their own KPI as their PV systems improve.

Commitment from national PV stakeholders and country ownership and empowerment are essential to sustain improvements and build a stronger AE reporting culture. Transparent and efficient communication and coordination between public health programs, EPI, and the national PV department is an important element for building functional PV systems, and centralized collection of post-marketing safety data is essential for safety signal detection and benefit-risk evaluations.

### WhatsApp LLC

WhatsApp is a registered trademark of WhatsApp Inc.

## Data Availability

The minutes of meetings and discussions held with national pharmacovigilance stakeholders or healthcare professionals during field visits and other data that are not personally identifiable information are available from the corresponding author on reasonable request.

## Abbreviations

ADR: Adverse drug reaction
AE: Adverse event
AEFI: Adverse event following immunization
DRC: Democratic Republic of Congo
EPI: Expanded Program on Immunization
HCF: Healthcare facility
HCP: healthcare professional
ICSR: Individual case safety report
KPI: Key performance indicator
LMICs: Low- and middle-income countries
MoH: Ministry of Health; NRA national regulatory authority
PIDM: Program for International Drug Monitoring
PV: Pharmacovigilance
SARS-CoV-2: Severe acute respiratory syndrome coronavirus 2
SSA: Sub-Saharan Africa
WHO: World Health Organization
